# Genotype vs. Phenotype and the Rise of Non-Communicable Diseases: The Importance of Lifestyle Behaviors During Childhood

**DOI:** 10.7759/cureus.458

**Published:** 2016-01-13

**Authors:** Brian W Wu, Paula M Skidmore, Olivia R Orta, James Faulkner, Danielle Lambrick, Leigh Signal, Michelle A Williams, Lee Stoner

**Affiliations:** 1 Keck School of Medicine, USC; 2 Department of Human Nutrition, University of Otago; 3 Department of Epidemiology, Harvard School of Public Health; 4 School of Sport and Exercise, Massey University; 5 Institute of Food Nutrition and Human Health, Massey University; 6 Sleep/Wake Research Centre, Massey University

**Keywords:** non-communicable diseases, physical activity, nutrition, obesity, sleep, genotype, phenotype

## Abstract

Despite continued research and growing public awareness, the incidence of non-communicable diseases (NCD) continues to accelerate. While a person may have a genetic predisposition to certain NCDs, the rapidly changing epidemiology of NCDs points to the importance of environmental, social, and behavioural determinants of health. Specifically, three lifestyle behaviours expose children to important environmental cues and stressors: physical activity, nutritional intake, and sleep behaviour. Failure to expose children to proper gene-environment interactions, through the aforementioned lifestyle behaviours, can and will predispose children to the development of NCDs. Reengineering the environments of children can induce a paradigm shift, from a predominantly biomedical health model of treating symptomology, to a more holistic model based on encouraging appropriate behavioral decisions and optimal health.

## Editorial

### Introduction

Globally, not only is the incidence of non-communicable diseases (NCDs) accelerating, but they are increasing among younger age groups [1]. For example, obesity, which is now seen in over 42 million children worldwide, increases the risk of premature onset of subsequent illnesses, including musculoskeletal disorders, some cancers, and Type 2 diabetes [2]. The earlier acquisition of cardiometabolic disease risk factors and the earlier onset of NCDs is resulting in a prolonged and enhanced burden of disease. In addition, it is threatening the economic development of low and middle-income countries least prepared to manage chronic medical conditions [3].

While a person may have a genetic predisposition to certain cardiometabolic disorders or NCDs, the rapidly changing epidemiology of NCDs points to the importance of environmental, social, and behavioural determinants of health. Further, investigators have begun to emphasize the importance of the interplay of genetic and environmental cues during early childhood development (during critical periods of development) when biological systems are most alterable or plastic, and before chronic disease risk trajectories are hard-wired. Important environmental cues come in the form of physical activity, nutritional intake, and sleep behaviour [4]. Failure to expose children to proper gene-environment interactions can and will predispose children to the development of NCDs.

### Physical activity

The World Health Organization (WHO) recommends that children aged 6-17 years participate in at least 60 minutes of moderate-to-vigorous intensity physical activity (MVPA) every day [5]. Eustress, in the form of MVPA, provides the environmental cues required for optimal human development and function. Considering the overall genetic makeup of Homo sapiens has remained similar for the last 10,000 years, the human genome has been programmed for a physically active, hunter-gatherer lifestyle [6]. Furthermore, the genome has evolved to be dynamic; to ensure observable characteristics (i.e., phenotypic characteristics, such as cardiorespiratory fitness or fat and lean muscle mass) are best suited to the particular environment. As a result of adequate physical activity, skeletal muscle fibers thicken, the cardiac muscle becomes stronger, the arterial system becomes more elastic, and bones thicken [7]. Furthermore, one of the most important mechanisms of physical activity is to increase the sensitivity of skeletal muscle but not fat cells to insulin, promoting fat uptake into skeletal muscles rather than around the abdomen [8].

The recent 2014 United States Report Card of Physical Activity for Children and Youth estimates that only one-quarter of children (6-15 y) in the United States meet WHO physical activity recommendations [9]. Similar findings are available in many countries making childhood and youth physical inactivity a global concern [10]. The result is that today’s children are not only becoming pre-conditioned for NCDs but are also, due to developmental issues, unable to achieve the level of health achieved by their parents [11]. Physical inactivity permits the development of various kinds of NCDs, including obesity, Type 2 diabetes mellitus, and cardiovascular disease [12-13], even in children and adolescents [14]. A recent comprehensive analysis of the effects of physical inactivity on the global burden of NCDs and mortality estimated that 6% of coronary heart disease cases, 7% of Type 2 diabetes, 10% of breast and colon cancers, and 9% of deaths are directly attributable to physical inactivity [15]. Arguably, the best way to tackle these NCDs is to support primary disease prevention and health promotion efforts at population levels, specifically, in ways that will ensure that children are provided with the educational and environmental resources necessary for promoting optimal health and development across the life course.

### Nutrition

Current WHO guidelines stress the importance of increasing diversity and intake of animal-source foods (milk, eggs, and lean cuts of non-processed meats), focusing on high-quality, nutrient-rich foods [16]. From an evolutionary standpoint, the digestive and metabolic capabilities of the body have evolved to handle the specific types of foods that the WHO describes, including foods high in protein and fiber. Protein is necessary for tissue development and fiber slows digestion, allowing carbohydrates to be properly absorbed. The eustress from this diet allows the body to properly metabolize, convert, and deliver the nutrients and energy needed for development [17]. These nutrients include calcium and vitamin D, iron, vitamin A, folate, and iodine, all of which are classified as important for bone growth, vision, and cognitive development in children [16].

The WHO estimates that 170 million children are overweight or obese, and excess caloric intake from unhealthy foods is an important contributing factor [2]. Furthermore, it is believed that this consumption of unhealthy foods is the cause of at least 14 million deaths or 40% of all deaths each year from NCDs [18]. Modern diets increasingly consist of processed and unhealthy foods, which are stripped of vital micronutrients, protein, and fiber. Such changes fundamentally alter how the digestive and metabolic system function; evolutionarily, the body is designed to optimally maintain energy levels through minimizing glucose levels in the bloodstream and storing energy as glycogen [17]. However, the intake of excessive unhealthy foods overwhelms the liver, compromising the conversion of energy to glycogen by the liver. As a result, this energy is stored as fat, often in the form of visceral fat, leading to metabolic complications [19-21]. Moreover, children who are exposed to poor quality food sources are not provided with the nutrition required to promote a physically active lifestyle [22], compounding the risk for the development of NCDs.

### Sleep behaviors

Sleep is a behavioral strategy that evolved to optimize the efficiency of a number of biological functions. Proposed theories for why sleep has been adapted include: 1) the resulting safety features surrounding inactivity during times of danger, 2) reductions in energy demands and conservation of resources, 3) restoration of tissues and physiologic systems, and 4) the organization of brain structures during times of plasticity in infants and young children [23-24]. The National Sleep Foundation (NSF) recommends 7 to 8 hours of sleep for adults, and 10 to 12 hours for children aged 5-12 years [25]. Sleep insufficiency compromises alertness, productivity, memory and information consolidation, mental health, and physical activity; it is also associated with behavioral problems in children [26-28]. Recommendations from the NSF include the establishment of consistent sleep patterns, conducive sleep environments, and physical activity [25]. A life-course approach to chronic disease prevention is rooted in the idea that factors during the prenatal period, infancy, and early childhood, such as sleep, may determine the risk of NCDs later in life [29].

Sleep deprivation has short and long-term effects on health, and emerging evidence documents the association of short sleep duration with childhood obesity [30-31]. Plausible mechanisms for the effects of sleep on weight gain include disruptions in the normal functioning of appetite regulation as well as the endocrine and autonomic systems [23]. Other harmful consequences to sleep insufficiency are hyperactive sympathetic activity, elevated cortisol levels, hypoadiponectinemia, and insulin resistance [23, 32]. Furthermore, recent evidence links sleep efficiency to subsequent daily physical activity in women [33]. Although these results have not yet been documented in children, when coupled with the known effects of poor sleep behavior on appetite regulation and metabolism, this serves as additional evidence for the role of sleep deficiency in NCD pathology. 

### Conclusions

Prevention science, the systematic application of scientific methods directed towards disease prevention and health promotion, has yet to receive the full support necessary to tackle the growing NCD burden [34]. Within the U.S., between 2000 and 2011, the cost of treating NCDs was estimated to exceed 80% of the annual healthcare expenditure, whereas only 3% was spent on disease prevention programs [35]. This is despite the fact that NCDs are estimated to be the primary driver of the national debt over the next four decades [36]. Low levels of investment in prevention science represent a missed opportunity to (1) promote optimal child health and development, and (2) mitigate the rising tides of NCD burden. This bodes the question: If there is greater investment in prevention science, where should this investment be primarily targeted?

Currently, the National Institutes of Health in the U.S. allocates approximately 20% of its annual $30 million budget to disease prevention, of which less than 10% is spent on human behavioral interventions and modifiable risk factors [37]. Not only should a greater allocation be applied to behavioral interventions, but we argue that there should be a specific focus on children. Arguably, since NCDs tend to result from gene-environment interactions, and we cannot re-engineer our genes (at least not yet), more emphasis should be placed on re-engineering our environments. While environmental re-engineering may be controversial when applied to adults, due to the removal of choice/freedom, it is relatively uncontroversial to regulate the environments of children. More emphasis on implementing public health programs at multiple levels should be required for all organizations. These levels include prenatal care, primary care education initiatives, school-based physical education, sleep education, healthy food options, community-based support groups, government-led public media campaigns, and regulations and taxes for sweetened beverages. Such a multi-level investment will enable a paradigm shift, from a predominantly biomedical health model based on treating symptomology, to a more holistic model based on encouraging appropriate behavioral decisions and optimal health.
